# Mapping the Drivers of Multisectoral Nutrition Governance and Its Link to Nutrition Outcomes in Kenya: A Qualitative Inquiry

**DOI:** 10.3390/nu17020209

**Published:** 2025-01-08

**Authors:** Jacob Korir, Wanjiku N. Gichohi-Wainaina, Oak-Hee Park, Sung-Wook Kwon, Malinda J. Colwell, Wilna Oldewage-Theron

**Affiliations:** 1 Department of Nutritional Sciences, College of Health and Human Sciences, Texas Tech University, Lubbock, TX 79409, USA; jkorir@ttu.edu; 2 WorldFish, Jalan Batu Maung, Bayan Lepas 11960, Penang, Malaysia; 3 Department of Interdisciplinary Human Sciences, College of Health and Human Sciences, Texas Tech University, Lubbock, TX 79409, USA; 4 Department of Political Science, College of Arts and Sciences, Texas Tech University, Lubbock, TX 79409, USA; 5 Department of Human Development and Family Sciences, College of Health and Human Sciences, Texas Tech University, Lubbock, TX 79409, USA

**Keywords:** multisectoral nutrition governance, Kenya, multisectoral nutrition approaches, nutrition policy, nutrition programming

## Abstract

Background: Malnutrition remains a significant public health issue in Kenya. Multisectoral Nutrition Governance (MNG) is increasingly being acknowledged as a catalyst for enhancing nutrition programming and outcomes. Effective MNG establishes policies, systems, and mechanisms that enable coordinated, adequately funded, and sustainable nutrition actions across sectors; however, its understanding and progress assessment remain inadequate. Objective: This study aimed to qualitatively assess the status of MNG and propose strategies to strengthen MNG mechanisms for improved nutrition actions and outcomes in Kenya. We hypothesized that effective performance across the MNG domains is associated with effective multisectoral nutrition actions and improved nutrition outcomes. Design: This study used a qualitative design to assess the MNG status over the past 10 years (2012–2023). Nineteen program managers and officers from government and non-governmental institutions implementing nutrition at the national level were included. Data collection was conducted between January and March 2024 through key informant interviews (KIIs). Thematic analysis, guided by both inductive and deductive coding, was carried out using MAXQDA (Maximizing Qualitative Data Analysis) software. Results: The findings indicate progress in strengthening MNG in the previous decade, though gaps persist. The progress was driven by improved political awareness and commitment, the adoption of nutrition policy and planning frameworks, and improved coordination. Constraints that impede MNG progress include inadequate financing and over-reliance on donor funding, limited translation of commitments to actions, lack of unified monitoring and evaluation (M&E) systems and fragmented policies. Conclusions: Strengthening multisectoral M&E systems that allow timely collection and utilization of data, ensuring sustainable financing for nutrition, enhancing accountability mechanisms and improving coherence across sectors are important for further improvement of MNG.

## 1. Introduction

Malnutrition continues to be an important public health issue in Kenya. Some nutrition indicators such as stunting and underweight have improved in recent years, largely due to efforts across various sectors such as health, education, agriculture and social protection. However, indicators such as micronutrient deficiencies and overweight, obesity, and diet-related Non-Communicable Diseases (NCDs) remain high, especially in vulnerable populations in the country [1,2,3]. Malnutrition poses long-term implications on health, cognitive development, and economic productivity [4,5].

Multisectoral Nutrition Governance (MNG) is increasingly recognized as an enabler of improved nutrition outcomes in Kenya and other developing countries [6,7,8,9]. Effective MNG ensures that political commitment is sustained, leadership across sectors is robust, and essential resources, including financing and human capacity, are aligned toward common nutrition goals [10,11]. The interplay of these factors within and across various sectors highlights the complexity of achieving MNG [12,13]. Despite the global recognition of MNG approaches as essential for addressing malnutrition, empirical evidence on the operationalization of MNG in complex contexts such as Kenya remains limited [12]. The Kenyan context presents unique challenges, including decentralized governance structures, diverse socioeconomic conditions, and competing priorities across sectors, which collectively complicate the implementation of MNG strategies. While studies in countries such as Ethiopia, Ghana, and Nepal have explored various dimensions of MNG, research focusing specifically on the Kenyan context is inadequate [8,13,14,15]. Addressing this gap is essential to enhance the understanding of MNG in Kenya and inform tailored strategies. Additionally, the absence of standardized metrics or frameworks for assessing MNG impedes routine evaluation and comparative analysis across contexts [16,17]. This study aims to address these critical gaps by providing evidence on the current status of MNG in Kenya, identifying key enablers and challenges, and proposing a framework for the qualitative assessment of MNG, along with actionable opportunities for improvement. The study thereby contributes to advancing the understanding of MNG in developing countries and fostering evidence-based policy and programmatic actions.

The success of multisectoral approaches and nutrition governance depends on the establishment of enabling structures, such as the development and implementation of national nutrition policies and plans. In Kenya, frameworks like the National Food Security and Nutrition Policy (NFSNP) and the Kenya National Nutrition Action Plan (KNNAP) have laid the foundation for MNG. These frameworks ensure clear role delineation, integration of nutrition into sectoral plans, provision financing and establishment of tracking mechanisms [2,18,19]. However, the presence of policies and plans alone is insufficient; political goodwill, adequate financial investments, and the capacity to monitor and evaluate progress are equally critical. Without these supporting pillars, the potential of effective MNG remains unrealized, limiting its effectiveness in addressing nutrition challenges [7,13,20]. Given the pivotal role of MNG in ensuring good nutrition outcomes, it is important to investigate the barriers, enablers, and opportunities that shape its effectiveness in Kenya. This, however, remains a challenge considering that nutrition is implemented across various sectors and there is a lack of clarity on who is responsible for the overall monitoring of MNG. Furthermore, actors lack mechanisms to evaluate how effectively they are implementing MNG and the recognition of MNG’s importance varies across sectors. Assessing MNG requires a clear understanding of both the key components that constitute MNG as well as applicable principles. MNG is defined as the iterative process of decision-making, policy development, strategy implementation, and monitoring aimed at improving nutrition outcomes across various sectors and levels. MNG encompasses six domains: (i) political commitment, authority and leadership, (ii) policy coherence and coordination, (iii) transparency and accountability, (iv) financing, (v) capacity and capability to implement nutrition actions, and (vi) results measurement and monitoring [7,12,21]. The definition of MNG aligns closely with the principles of collaborative governance, which emphasize the active involvement of multiple sectors, stakeholders, and institutions through formal, coordinated, consensus-oriented, and deliberate processes to address complex public policy challenges [22,23]. Collaborative governance highlights key domains or dimensions of MNG such as leadership, coordination, collaboration, and accountability. Applying this theoretical lens allows us to examine how different actors and sectors work together to effectively plan and implement nutrition policies and programs. The study therefore aimed to qualitatively assess the status of MNG and propose strategies to strengthen MNG mechanisms for improved nutrition outcomes in Kenya. We hypothesize that good performance across the MNG domains is associated with effective multisectoral nutrition actions and improvements in nutrition outcomes. We aimed to answer the following research questions: (i) what is the current MNG status and how has it evolved in the last 10 years? (ii) What are the enablers, opportunities, gaps, and barriers of MNG? (iii) How does change in MNG over time influence nutrition outcomes? and (iv) how can MNG approaches be enhanced in Kenya?

## 2. Materials and Methods

### 2.1. Study Design

This study was nested within a larger study designed to assess MNG and its drivers within the Kenya’s national nutrition landscape. This study used a qualitative design to assess the status of MNG over the past 10 years (2012–2023). The design was chosen to explore the perceptions and experiences of key nutrition stakeholders, providing rich, contextual insights into MNG. The two-time points allowed the assessment of temporal shifts and trends in MNG over time and provided valuable insights into the evolution of MNG frameworks, enabling the identification of trends, progress, and challenges. The two key frameworks, NFSNP and the first KNNAP, were adopted in 2012 marking a significant milestone.

### 2.2. Study Participants and Sample Size

This study was conducted by executing key informant interviews (KIIs) among respondents working in eligible ministries or sectors at the national level. Eligible ministries or sectors comprised those whose core functions, policies, or plans directly aligned with Kenya’s nutrition improvement agenda [2,19,24]. In this study, the terms ‘ministry’ and ‘sector’ were used as related terms due to their interconnected roles and players in Kenya. While a ministry refers to a government body responsible for policy formulation and oversight, sectors such as education or agriculture represent broad thematic areas often led by specific ministries but involving multiple stakeholders within the same area [25].

Participants were mapped with the support of the Division of Family Wellness, Nutrition and Dietetics. A strict inclusion-exclusion criteria was followed to ensure that participants who have in-depth knowledge, experiences and perspectives on MNG were selected. The following inclusion criteria were applied: (i) government staff who were working as program managers and officers in a department, division, or unit related to nutrition in the nine eligible ministries/sectors mapped for this study. The eligible ministries were Health, Agriculture, Education, Social Protection, Water and Sanitation, National Treasury and Economic Planning, Public Service, Gender and Affirmative Action, Trade, Investments and Industry, and Devolution and Planning. In the Kenyan government context, a program manager oversees multiple projects and aligns goals with organizational objectives, while a program officer implements specific components. Their closely linked roles ensure cohesive program planning execution, which are both relevant for the study [25]. (ii) Program managers and officers from non-governmental institutions/directly supporting the nutrition sector at the national level to plan and implement nutrition actions. The exclusion criteria were as follows: (i) government staff from ineligible ministries (i.e., Defense, Energy, Foreign Affairs, Tourism and Wildlife, Information and Communication, Environment and Forestry, Mining and Blue Economy, East African Community, Youth Affairs and Sports, and Interior and National Administration). (ii) Government staff from eligible ministry departments that play no direct role in nutrition planning and programming, e.g., administration, human resources, and legal services. (iii) Program managers and officers from non-governmental institutions that do not directly support the nutrition sector at the national level. In this study, respondents’ characteristics such as ethnic groups and education attainment were not assessed, as the focus was on institutional and policy-level dynamics rather than demographic subgroup analysis. Additionally, collecting sensitive data could reduce response rates given the limited relevance to the study objectives. Using this approach resulted in a sample of 19 respondents (13 female and 6 male), which exceeded the KII threshold of 12, the acceptable level of saturation for qualitative KII research [26].

### 2.3. Data Collection Tool and Process

The study was conducted by a team of experts in nutrition sciences, public health, and qualitative research. The team included two researchers with advanced degrees and extensive experience in qualitative studies in nutrition sciences, and three members trained in mobilization, interviewing, transcription and qualitative data analysis. A semi-structured interview guide was used to collect the data (Appendix A.1). The questions covered the six MNG domains and were relevant for both program managers and officers in government and non-governmental institutions. Eligible respondents were invited to participate through individual communication via email and telephone. The interview guide was administered in virtual interviews conducted in English between January and March 2024. In addition to interview questions, participants were asked contextual/background characteristics questions such as their roles, and the specific government ministries they worked with or supported among others.

Expert review and pre-test approaches were applied to ensure the reliability of the interview guide. Two experts who are conversant with Kenya’s nutrition context were invited to review the tools and comment on whether the questions and prompts adequately capture participants’ perceptions and experiences on MNG. Secondly, the tool was pre-tested on one government staff member and one non-governmental stakeholder who were not part of the sampled respondents [27]. In addition to the two steps, other procedures that improve the reliability of qualitative tools recommended by Long and Johnson (2000) such as data triangulation, thorough record keeping, and inclusion of rich verbatim descriptions were adhered to [28]. To ensure anonymity while maintaining the ability to attribute perspectives consistently, each respondent was assigned a generic identifier (e.g., KII ID 01) in addition to their sector and role (e.g., health sector, program manager).

### 2.4. Data Analysis

The study team transcribed audio recordings verbatim for each interview. Each transcript was then double-checked for accuracy by listening to the audio while going through the transcripts to ensure no information was omitted. Data management and analysis were conducted using MAXQDA version 24 (VERBI Software, Berlin, Germany), given its robust capabilities for organizing and coding of qualitative data systematically [29]. MAXQDA facilitated the development of the code book and application of codes in one interface allowing for efficient identification of patterns across the data. The deductive coding approach was primarily employed and complemented by inductive coding to analyze data into key themes, concepts, and categories. The deductive coding approach was guided by the predefined research objectives and questions, allowing the team to apply codes aligned with key thematic areas of interest. In parallel, the inductive coding approach facilitated the identification of emergent codes and themes from the data. This hybrid approach was adopted to balance the need for structured analysis while capturing emerging themes [30].

The coding process involved multiple steps to ensure rigor and reliability. An initial codebook was developed collaboratively by the research team, with definitions and examples for each code to promote consistency. Each transcript was independently coded by two team members to establish inter-rater reliability, with discrepancies resolved through group discussion and consensus. Regular team meetings were held to refine the codebook and adapt it based on iterative coding rounds. MAXQDA’s features, such as memoing and code visualization, were leveraged to facilitate pattern identification and thematic development across the transcripts.

Thematic analysis was employed to systematically identify, analyze, and interpret patterns within the data, focusing on key codes/key concepts related to MNG. Coding was conducted at the paragraph level to capture broader contextual meanings and patterns within the data. An example of the code was ‘positive changes in commitment to improve multisectoral nutrition approaches in a specific ministry’. Recurring related codes were grouped into subthemes such as the ‘the level of political commitment in addressing nutrition issues, institutional support provided to staff, and level of multisectoral nutrition integration.’ The nine subthemes were synthesized to six overarching themes to ensure a comprehensive understanding of the data (Table 1) [30]. Key secondary sources including the NFSNP, KNNAP, and sector strategic plans were used to corroborate the data where necessary. This process ensured that interpretations were grounded in both primary and contextual evidence. The themes provided a framework for assessing the status of MNG, its evolution over time, successes, challenges and its link to nutritional outcomes.

### 2.5. Ethical Approval and Consent

Ethical approval was obtained from the Texas Tech University (TTU) Institutional Review Board (IRB), approval number IRB2023-493, and Amref Ethics and Scientific Review Committee (ESRC), approval number ESRC P1443/2023. A research permit was obtained from the National Commission for Science, Technology, and Innovation (NACOSTI) license number NACOSTI/P/23/27685. In addition, a letter of support to conduct the study was provided by the Office of the Director General, Ministry of Health Kenya, number Ref MOM/ADM/1/1/82/361. Informed consent was sought from all respondents to collect and publish the data.

## 3. Results

### 3.1. Respondents’ Characteristics

Table 2 presents the respondents’ characteristics. More respondents (*n* = 11, 57.9%) worked for the government compared to those who worked for non-government organizations (*n* = 8, 42.1%). The majority (*n* = 13, 68.4%) of the respondents were program managers. Most respondents (*n* = 16, 88.9%) worked or supported primary line ministries (Health, Agriculture, Education and Social Protection). The health sector had the highest representation (*n* = 7, 36.8%) compared to other sectors. The dominance of the Health ministry reflects the centrality of nutrition coordination within the Health ministry and the availability of health sector participants for the KIIs.

### 3.2. Synthesis of Key Findings Across the Themes and Subthemes

The analysis and synthesis identified multiple instances of consensus (agreement or disagreement) across subthemes as summarized in Table 3. This offered critical insights into progress or stagnation across various MNG domains, highlighting areas for targeted improvement. To prevent inflation of frequencies, we documented the number of unique respondents contributing to each code, acknowledging that some respondents either lacked information on certain topics or chose not to discuss them. We employed frequency translation terms, defined as follows: ‘virtually all’ (>90%), ‘most’ (60–89%), ‘several’ (30–59%), and ‘few’ (<30%), to represent the prevalence of perspectives across respondents. Additionally, representative quotes are presented under each subtheme to provide context and enrich the interpretation of the findings.

#### 3.2.1. Current Status of MNG and How It Has Evolved in the Last 10 Years

Several of the respondents agreed that Kenya has made progress in enhancing MNG in the past decade. The respondents highlighted improved nutrition policy frameworks and enhanced coordination across multiple sectors among other developments. The respondents highlighted that national policies and plans, such as the KNNAP and the NFSNP, have been instrumental in enhancing coordinated nutrition actions across various sectors such as health, agriculture, education, and water, sanitation, and hygiene (WASH). However, some participants highlighted uneven progress in MNG, especially at the county level. Counties receiving support from partners have witnessed more progress and have developed and implemented County Nutrition Action Plans (CNAPs) whereas those not receiving support have struggled with CNAP development and resource allocation for nutrition. Most of the participants also highlighted the need for uniform progress across relevant sectors in MNG as opposed to focusing only on the health and agriculture sectors.

*“From the time NFSNP and KNNAP were rolled out we have seen improved multisectoral nutrition planning, action and goodwill across sectors implementing nutrition. Health, agriculture, social protection, and education among other ministries incorporate KNNAP actions in their annual planning and budgeting. However, most of the progress has been witnessed in the health and agriculture sectors.”*

(KII ID 018, health sector, program manager)

*“Health, nutrition and agriculture are devolved. Some county governments have been successful in domesticating KNNAP. They have developed CNAPs and they are implementing them with success, these are the ones mainly supported by partners. Counties with no partner support are struggling to develop and implement CNAPs.”*

(KII ID 05, health sector, program officer)

#### 3.2.2. Leadership, Coherence, and Institutional Support

Several participants of those who responded agreed that there is sufficient political commitment, authority and leadership to address nutrition challenges in Kenya. Those who disagreed emphasized that despite political leaders being aware of and advocating for improved nutrition, programmatic and financial commitments stipulated in national plans have not yet been effectively translated into concrete actions. For instance, the high-level nutrition coordination structures outlined in the NFSNP are yet to be operationalized, which has led to fragmentation. They also highlighted the lack of a unified and sustainable accountability mechanism that ensures that nutrition remains a priority amid transitions in leadership.

*“Nutrition rings in the minds of political leaders……they are excited when you discuss with them and develop plans but after that many go back to business as usual……therefore turning commitments to action is still a challenge.”*

(KII ID 09, health sector, program officer)

*“The food security and nutrition policy defined structures to improve multisectoral actions but structures are yet to be fully implemented. In addition, even if we have good plans and there is no adequate funding and capability then little will be achieved especially at the county level therefore we need to look at these things if we are to realize multisectoral nutrition governance.”*

(KII ID 15, agriculture sector, program manager)

Several respondents expressed optimism about the progress of nutrition policy coherence and multisectoral coordination in Kenya, despite ongoing challenges. They highlighted the positive impact of frameworks such as the KNNAP and CNAPs, which have provided structured guidelines for nutrition plans and actions. Additionally, platforms like the SUN movement, the National Interagency Coordination Council (NICC), and programs such as Nutrition and Economic Inclusion for Households (NICHE) have further enhanced cross-sectoral collaboration and coherence. However, respondents noted that while coherence and coordination are prioritized in national frameworks like the KNNAP and the NFSNP, these agendas are not yet fully integrated into sector-specific plans. This misalignment is particularly evident in sectors like agriculture, health, and social protection, where competing objectives and varying mandates often hinder unified action toward reducing malnutrition.

*“We talk a lot about collaboration and coordination but we need to reflect more on coherence. Agriculture has the food security and nutrition policy, health has the health sector policy and then there is the KNNAP that is multisectoral and for all the other sectors. However, sectors cannot implement those activities because they don’t have a full mandate to implement, for example, nutrition-sensitive agricultural programs Therefore we need to critically look at how various policies and plans align with sector plans and mandates.”*

(KII ID 14, agriculture sector, program manager)

*“We are seeing specific programs covering more than one sector such as NICHE and National Information Platform for Food Security and Nutrition (NIPFN). The government is taking the lead in these programs both in their planning, implementation and coordination. The programs advance the vision of multisectoral policies at the national and county levels. However, we need to ensure that counties are supported to develop expertise for multisectoral nutrition actions.”*

(KII ID 08, health sector, program officer)

#### 3.2.3. Management Capacity and Financing

Participants who responded were evenly divided in their views on the level of capacity to plan and implement nutrition and the effectiveness of capacity-building initiatives for nutrition. Those who believed that nutrition capacity is improving highlighted the existence of a structured capacity development framework for nutrition, the integration of capacity-building objectives in the KNNAP, and various capacity-building initiatives targeting government and non-governmental staff. However, others expressed concerns about the lack of progress, noting that capacity-building efforts are often donor-driven and primarily focused on the health and agriculture sectors. Furthermore, they highlighted that there is an emphasis on the number of individuals trained rather than on evaluating whether the trained personnel are effectively applying the skills to enhance nutrition programming.

*“……There has been significant improvement in the technical capacity for nutrition……however it is not about nutrition knowledge only, we need to focus on building capacity for planning, evaluation, advocacy……capacity building has to be tailored to the needs and be led by the government.”*

(KII ID 16, health sector, program manager)

*“Capacity building is mostly defined by development partners as opposed to the needs which cause imbalances….so one week a nurse may attend one training and next week they are in another and they end up with no time to do their work……capacity development needs to be restructured and redefined and there is need to cover all the sectors. We also need to ensure that it is uniformly distributed in all carders.”*

(KII ID 04, health sector, program manager)

Most of respondents agreed there is insufficient domestic resource allocation to nutrition leading to over-reliance on external donor support, which is unsustainable. Although there are improvements such as earmarking budget lines for nutrition and the development of accountability scorecards for nutrition, the lack of consistent funding hampers the implementation of nutrition programs, especially in regions without donor support. Additionally, the respondents also indicated that the current allocation is largely used to purchase nutrition commodities and respond to emergencies such as drought. They emphasized the need for the government to allocate a minimum fixed budget for nutrition and to strengthen financial tracking mechanisms to build a stronger case for sustained investment in nutrition.

*“Budget to nutrition from the government is grossly below what is expected……we have seen improvement setting up budget lines for nutrition but the majority of funds are used to purchase [nutrition] commodities and respond to emergencies……there is a lot of dependence on partners. Counties that are not supported by partners have major gaps.”*

(KII ID 18, health sector, program manager)

*“Yes, budgets very low but we need to evaluate how we track commitments and allocations……I feel that nutrition-sensitive allocations are not adequately tracked……we also need to ask ourselves how we can ring-fence minimum allocations from government so that so that funding is sustained even when political leadership change……this needs a strong investment case for nutrition.”*

(KII ID 04, health sector, program manager)

#### 3.2.4. Results Measurement, Monitoring and Accountability

Several participants agreed that there are adequate systems to collect, disseminate, and foster the utilization of nutrition data. While some respondents recognized advancements in the development of nutrition monitoring and evaluation frameworks, challenges still exist. This includes the lack of consensus on nutrition indicators to be tracked across sectors, inadequate inclusion of nutrition indicators in nationwide surveys and lack of timely analysis and use of data to inform programs. Other challenges highlighted by respondents include overemphasis on health information systems (nutrition-specific), the lack of a comprehensive nutrition-specific and nutrition-sensitive surveillance system that can track nutrition indicators across multiple sectors, and inadequate coverage of some population groups such as adolescents and the elderly.

*“A broad-based surveillance system that covers comprehensive nutrition indicators is lacking……the KDHS [Kenya Demographic Health Survey] covers some nutrition indicators but is unable to segregate drivers of malnutrition at the county and sub-county level. This means that the data is then not useful to inform programming at the county level.”*

(KII ID 12, education sector, program officer)

*“We have made progress in developing nutrition indicators and monitoring and evaluation plans……there are gaps in ensuring that the various systems in health, education, social protection are integrated and speak to each other…we also need to focus on other population groups such as adolescents as opposed to children under five, and strengthen nutrition surveillance……these are areas we need to advocate.”*

(KII ID 07, health sector, program officer)

In terms of accountability for nutrition, most participants who responded agreed that expectations are clear but there is a need to strengthen mechanisms that will foster accountability for nutrition commitments at various levels. They agreed that KNNAPs and CNAPs alongside nutrition scorecards that are being rolled out have clarified what is expected from various sectors. However, accountability should not rely solely on tools such as scorecards but also focus on effective monitoring and evaluation that enhances the timely collection and dissemination of data across sectors. Some participants pointed out that accountability needs to be broad-based from top-level officials down to community workers. They also highlighted that the placement of nutrition coordination at the division level makes it difficult to hold senior leaders accountable as compared to nutrition being at a higher administrative level.

*“There is clarity on what needs to be done to improve nutrition from the highest level to community volunteers and extension workers……the KNNAPs and CNAPs and nutrition scorecards have helped a lot……However it is hard to hold high-level leadership to account when nutrition is at division level……we need to advocate for the high-level placement of nutrition as stipulated in the [FSNP] policy.”*

(KII ID 04, health sector, program manager)

*“Accountability for nutrition is inadequate because there is focus on tools such as scorecards only……we need to start from generating and synthesizing data and disseminating information to the that is accurate and target correct audiences……We need to foster all-rounded accountability where accountability happens not only at the senior level but also at the implementation level.”*

(KII ID 04, health sector, program officer)

#### 3.2.5. Link Between MNG Status and Improved Nutritional Outcomes over Time

Few respondents were able to explain the relationship between MNG and improved nutrition outcomes in the last 10 years. Some acknowledged that while improved multisectoral actions, coordination, and commitment to nutrition governance have played a role in advancing nutrition indicators, such as reductions in stunting and underweight, the link remains complex. Respondents noted that various policies that are not directly aimed at improving nutrition (such as education and economic development policies) contributed to better nutrition outcomes; therefore, it is challenging to pinpoint the role of MNG. Additionally, while progress has been made in reducing undernutrition, challenges such as the rising prevalence of obesity and non-communicable diseases (NCDs) suggest that nutrition is influenced by multiple factors beyond MNG, including improvement in the economy, literacy, food systems, and universal healthcare among others. This complexity makes it challenging to isolate the impact of MNG from other contributing factors. They indicated that additional studies are required to understand the relationship between improvement in MNG status improvements.

*“I am not fully sure whether improvement in nutrition outcomes in the past 10 years be explained by the improvement in multisectoral nutrition governance alone. We have seen improvement in planning, coordination, prioritization and advocacy for nutrition in the past 10 years across sectors and this has led to improvement in nutrition indicators such as stunting and underweight but we still have challenges with the rising level of obesity and NCDs [Non-Communicable Diseases]. This is an indication that nutrition is influenced by many other factors such as the economy and education among other things. This means that nutrition governance will partially explain the situation therefore it is also important to assess other factors.”*

(KII ID 14, agriculture sector, program manager)

#### 3.2.6. Opportunities to Improve MNG

Virtually all respondents identified opportunities to enhance MNG across various domains. A key strategy emphasized by the respondents was ensuring that the KNNAP and CNAPs are aligned with sectoral policies and that nutrition is integrated into sectoral strategies, annual work plans, and budgets. Respondents emphasized the need to maintain nutrition as a priority on the government’s agenda and advocacy for adequate resources for nutrition, especially from domestic sources. The importance of strengthening cross-sectoral coordination and accountability mechanisms such as using the nutrition scorecards to monitor progress was mentioned. Similarly, respondents highlighted the need for a unified monitoring and evaluation system that encourages cross-sectoral collection and dissemination of information to enhance data-driven policy and programming decisions. This can build on ongoing efforts such as the Kenya Health Information Systems (KHISs) and the National Information Platforms for Food Security and Nutrition (NIPFN). Equally, participants pointed to the need for capacity-building efforts across all sectors, emphasizing not only technical skills but also the capacity for planning, tracking, and accountability for nutrition. Furthermore, capacity-building support efforts should extend beyond traditional sectors to include non-traditional sectors and counties. Lastly, some participants noted the need to advocate for nutrition’s elevation within high-level governance structures with convening powers in line with the NFNSP.

*“We need to put in place several things to ensure effective multisectoral nutrition governance is achieved in Kenya. First, the national nutrition action plan should integrate more nutrition-sensitive actions and ensure that the roles of different sectors is clearly delineated. Secondly, we need also to ensure that the non-traditional ministries and counties’ capacity is brought to a good level. In addition, we need to ensure that accountability in terms of scorecards is fully implemented to hold stakeholders accountable. Finally, we need more resources for nutrition, especially from domestic sources.”*

(KII ID 19, health sector, program manager)

*“We need to be united and come together as all sectors and implement the Kenyan national nutrition action plan and the food security and nutrition policy. We need to identify more champions and make sure that the information that we have is shared and is used to influence action ……in addition to advocacy for more resources for nutrition and positioning of nutrition at a high level of governance with convening powers……”*

(KII ID 08, health sector, program officer)

## 4. Discussion

The study assessed the status of MNG with the aim of proposing strategies to strengthen MNG for improved nutrition programming and outcomes in Kenya. The information collected across the MNG domains helped in answering the following questions: (i) what is the current MNG status and how has it evolved in the last 10 years? (ii) What are the enablers, opportunities, gaps, and barriers of MNG? (iii) How does change in MNG over time influence nutrition outcomes? (iv) How can MNG approaches be enhanced in Kenya? This section examines the four research questions about the respondents’ perspectives.

### 4.1. Current Status of MNG and How It Has Evolved

Kenya has made progress in enhancing MNG in the past decade mainly through enhanced policy and planning frameworks, improved political commitment and improved coordination despite some existing challenges. Similar improvements in MNG have been observed in countries like Nepal, Bangladesh, Ethiopia, and Uganda, where efforts have yielded positive results in improving nutrition. For instance, in Ethiopia and Nepal, improvements in MNG have been realized through enhanced multisectoral collaboration, political prioritization of nutrition, accountability, coordination, and monitoring across sectors [15,31]. While many studies highlight improvements in MNG, recent evidence also points to stagnation or challenges in this area. For instance, the World Bank reports that progress in reducing child undernutrition has plateaued globally, with a lack of consistent improvement in nutrition governance indicated as one of the reasons [21]. The improvement in MNG in Kenya and other developing countries reflects a growing recognition of the need for effective governance mechanisms that set a precedence for integrated strategies addressing malnutrition [32,33].

### 4.2. MNG Enablers and Barriers

Our study observed the positive influence of existing frameworks, improved awareness of the importance of nutrition, and enhanced coordination and collaboration across various sectors on MNG. Like the KNNAP, national policies and plans in countries like Bangladesh and Nepal have successfully integrated multisectoral approaches to address malnutrition through frameworks that coordinate efforts across different ministries [7]. Similarly, improved coordination and collaboration was also reported as an enabler for integrated multisectoral approaches in Ethiopia [15]. However, the effectiveness of these collaborations often depends on clear role delineation, effective leadership, and consistent funding, challenges that can undermine sustained progress in MNG even when robust frameworks exist [34]. Despite improved awareness of the importance of nutrition among leaders, this should be translated to institutional commitments and specific actions so that it can act as a buffer against shifting priorities [35]. To further enhance the impact of MNG, efforts should be directed at establishing clear accountability mechanisms and ensuring that policy frameworks like the KNNAP are adequately resourced and implemented at all levels. Sustained investment in these areas can build a resilient nutrition governance system that responds effectively to evolving nutrition challenges [36]. The findings highlight the importance of a coherent approach to governance that requires balanced improvements across all domains and sectors to ensure sustained nutrition outcomes [37,38].

The findings of this study align with broader trends in MNG, where limited funding, inadequate monitoring and evaluation as well as inadequate accountability on nutrition commitments remain significant hurdles. Similar challenges have been noted in countries like Nepal, Bangladesh, Ethiopia, and Uganda where multisectoral coherence and integration of nutrition into broader development frameworks were limited by insufficient resource allocation and limited political will [39,40]. In terms of inadequate nutrition financing and over-reliance on donor funding, studies on MNG in Sub-Saharan Africa, including Ghana and Uganda, emphasize similar challenges that compromise long-term program sustainability [13,41]. Nutrition programs reliant on donor funding often reflect donor interests rather than local priorities, which undermines local ownership and sustainability [15,42]. Previous studies also underscore the importance of policy coherence and the enactment of robust legal frameworks to sustain nutrition efforts [7]. However, institutionalization remains a slow and politically nuanced process, often impeded by competing interests [10]. The findings emphasize the need for stronger linkages between the national and county level, which has been identified as a way forward in similar contexts [18,34]. This can be achieved through improving accountability mechanisms, dedicated nutrition budgets, and integration of nutrition indicators into broader policy frameworks both at the national and county levels.

### 4.3. Link Between MNG and Nutritional Outcomes

While some research demonstrates a positive link between MNG and improved nutrition outcomes, most respondents in our study did not associate MNG with these outcomes due to its complexity. For instance, a study in Nepal found that higher scores on the Nutrition Governance Index (NGI) were linked to improvements in underweight and stunting [8]. Similarly, research in low- and middle-income countries (LAMICs) highlighted that effective multisectoral coordination can significantly reduce stunting, wasting, and underweight in children [43]. The respondents in the current study highlighted MNG-related improvements such as enhanced policy frameworks, commitment and coordination that contributed to improved nutrition that concur with previous studies [2,18]. However, broader socio-economic drivers such as increased GDP, literacy rates, and healthcare access make it challenging to isolate the specific impact of MNG on nutrition outcomes. Further research is therefore needed to clarify the association between MNG and nutrition outcomes given the multifaceted nature of the factors involved.

### 4.4. Strategies to Enhance MNG Approaches

The opportunities for enhancing MNG align with other studies that highlight the importance of integration of nutrition in sectoral policy frameworks, sustained political commitment, adequate financing, enhanced accountability and streamlined M&E system for evidence-driven decision-making. Burkina Faso, Nepal and Zambia have demonstrated that having well-defined multisectoral nutrition plans aligned to sectoral plans can lead to increased prioritization and resource mobilization for nutrition [39,44,45]. Studies from countries like Ethiopia have demonstrated that expanding multisectoral platforms, such as technical working groups and national nutrition coordination bodies, plays a vital role in aligning objectives of different sectors, integrating diverse stakeholders and ensuring sustained political commitment. Similarly, the Seqota Declaration in Ethiopia, a high-level commitment to alleviate malnutrition, has facilitated the championing of high-level commitment, prioritization and financing for nutrition across sectors [15,46].

While other countries have experienced similar challenges, the level of emphasis on political stability varies. Kenya’s political context highlights the importance of resilience to political transitions, a finding distinct from studies in countries like Ethiopia and Senegal, where the focus has been more on operationalizing multisectoral mechanisms. Institutionalizing MNG through legal and policy frameworks could mitigate disruptions caused by political changes, ensuring continuity and stability. For instance, embedding MNG within Kenya’s existing development frameworks, such as the Vision 2030, could safeguard progress despite political shifts [10].

Operationalizing M&E mechanisms that are responsive to sectoral priorities and are adaptable to changing contexts is critical for sustaining MNG progress. Clearly defined roles and alignment across sectors are essential given that various sectors contribute to nutrition outcomes using different approaches, which often work in isolation. For instance, the Nutrition Enhancement Program in Senegal established clear definitions of sector roles, which significantly contributed to effective multisectoral governance [47]. The emphasis on cross-sectoral evidence-driven decision-making is also echoed in research from India, where data-driven nutrition policy decisions have bolstered implementation across sectors [17]. Additionally, aligning sectoral objectives under a common nutrition results framework can help address fragmentation and ensure sustained progress, even amid political transitions [48,49].

Countries such as Brazil have observed increased sustainability and improved outcomes through increased resource allocation for nutrition programs and embedding MNG into broader development policies [34]. Furthermore, Kenya can learn from countries such as Malawi, which is in the process of implementing multisectoral financial and programme indicator tracking mechanisms, aimed at enhancing commitment and accountability for nutrition financing and results at the national and sub-national levels [50]. Kenya’s reliance on donor funding remains a challenge, underscoring the need for domestic resource mobilization. Strategies such as ring-fencing (securing minimum fixed budgetary allocation from the government) budgets for nutrition both at the national and county levels could reduce external dependencies and enhance local ownership.

The way forward, as suggested by various studies, lies in improving domestic financing for nutrition, creating more robust M&E and accountability mechanisms, and reducing reliance on external donors [48,51,52]. Kenya can leverage such approaches but must adapt them to its devolved governance system, which requires coordination between national and county governments.

**Strengths and Limitations of the Study:** Our study addresses a critical gap in the literature by contributing to growing evidence on the status and factors associated with MNG. A strength of our study is its ability to capture in-depth, context-specific insights on the status of MNG across various sectors over time. Furthermore, MNG was broken down into constituent domains. This approach allows for a nuanced understanding of how political, institutional, and structural factors influence MNG, which quantitative studies may overlook. While this study achieved the requisite saturation level, participation relied on the availability and willingness of respondents to participate which may have led to response bias. Two out of the nine eligible sectors were not represented; however, this did not affect the results since all the primary sectors (health, agriculture, education and social protection) were represented. Additionally, the findings may have limited generalizability due to the specific focus on Kenya’s unique governance context, which might differ from other countries with different political, social, or economic contexts. Another limitation is that our findings are primarily applicable at the national level, as the MNG landscape and contexts vary across the 47 counties. Nevertheless, the national-level approaches do largely affect the sub-national since the two levels of government work closely in policy formulation and implementation.

## 5. Conclusions

The findings show progress in improving MNG in Kenya albeit some challenges remain. The drivers of improvement in MNG include enabling policy frameworks, improved political commitment and improved coordination. However, despite these advances, fragmented policies, limited translation of commitments to action, lack of integrated M&E systems and financial constraints continue to impede MNG. Areas that require improvement include enhancing integrated M&E systems that will allow timely collection and utilization of data, enhancing sustainable financing for nutrition, and elevation of nutrition to a high level of governance with convening powers. While the link between improved MNG status and nutritional outcomes is positive, further research is needed to better understand complex contextual factors and dynamics influencing this relationship. Future research could include additional stakeholders, such as local leaders and the public, to provide a more comprehensive understanding and implementation of MNG recommendations. In addition, future research and initiatives should focus on addressing the fragmented understanding and tracking of MNG, as implementation challenges frequently arise from insufficient awareness of MNG and its progress. Our study adds valuable knowledge that is essential for enhancing MNG for improved nutrition programming and outcomes in Kenya.

## Figures and Tables

**Table 1:** Qualitative description of the study themes.

Theme	Subtheme
**1. Current status of MNG and how it has evolved in the last 10 years**—The structure and functioning of MNG in Kenya and how it has changed in the past 10 years.	1. Progress across various domains of MNG over the past decade
**2. Leadership, coherence and institutional support**—The level of political commitment in addressing nutrition issues, institutional support provided to staff, and level of multisectoral nutrition integration, coherence and coordination.	1. Level of political commitment, authority and leadership to address nutrition challenges 2. Level of nutrition policy coherence and multisectoral coordination
**3. Management capacity and financing**—Inclusion of nutrition in the planning and budgeting processes and level of budgetary allocation to nutrition. It also includes the level of technical capacity and effectiveness of capacity-building initiatives.	1. Budgetary allocation to nutrition 2. Level of capacity to plan and implement nutrition, and capacity-building initiatives for nutrition
**4. Results measurement, monitoring, and accountability**—Quality of existing data collection, dissemination and utilization systems. It also includes clarity of roles at various levels and follow-up of nutrition commitments.	1. Status of systems to collect, disseminate and foster the utilization of nutrition data. 2. Clarity of expectations and status mechanisms to foster accountability for nutrition commitments
**5. Link between MNG status and improved nutritional outcomes over time**—Relationship between MNG and nutrition outcomes in the last 10 years (since the adoption of NFSNP and the first KNNAP in 2012).	1. Relationship between MNG and nutrition outcomes in the last 10 years.
**6. Strategies to enhance MNG approaches**—Opportunities to enhance MNG for effective nutrition programming and improved nutrition outcomes in Kenya.	1. Opportunities to improve MNG in Kenya

**Table 2:** Respondents’ characteristics.

Characteristic	Category	*n*/N	Percent
Respondent’s sex	Male	13/19	68.4%
	Female	6/19	31.6%
Government or non-government respondents	Government	11/19	57.9%
	Non-government	8/19	42.1%
Respondent’s role	Program managers	13/19	68.4%
	Program officers	6/19	31.6%
Category of respondent’s ministry ^a^	Primary	14/19	73.7%
	Secondary	5/19	26.3%
Ministry respondents work for or support	Health	7/19	36.8%
	Agriculture and Livestock	4/19	21.1%
	Education ^b^	3/19	15.8%
	Labor and Social Protection	1/19	5.3%
	Water and Sanitation	2/19	10.5%
	Treasury and Economic Planning	1/19	5.3%
	Devolution and Arid and Semi-Arid Lands (ASALs) ^c^	1/19	5.3%

^a^ Primary line ministries—Health, Agriculture and Livestock, Education, Labor and Social Protection. Secondary line ministries—Water and Sanitation, Treasury and Economic Planning, and Devolution and ASALS. ^b^ Includes academic staff from public and private universities. ^c^ Includes the Council of Governors representatives.

**Table 3:** Themes, subthemes and corresponding frequencies.

Theme	Subtheme	Frequency
1. Current status of MNG and how it has evolved in the last 10 years	There has been consistent progress across various domains of MNG over the past decade	15/19 (most)
2. Leadership, coherence, and institutional support	There is sufficient political commitment, authority and leadership to address nutrition challenges in Kenya	8/17 (several)
	There is adequate nutrition policy coherence and multisectoral coordination	11/19 (several)
3. Management capacity and financing	Nutrition is allocated sufficient budget	3/18 (few)
	There is sufficient capacity to plan and implement nutrition, and capacity-building initiatives for nutrition are impactful	10/19 (several)
4. Results measurement, monitoring, and accountability	The systems to collect, disseminate and foster the utilization of data are adequate	8/17 (several)
	The expectations are clear and there is a mechanism to foster accountability for nutrition commitments	15/19 (most)
5. Link between MNG status and improved nutritional outcomes over time	We can see a relationship between MNG and nutrition outcomes in the last 10 years (when NFSNP and first KNNAP were adopted)	3/9 (few)
6. Opportunities to enhance MNG approaches	Several opportunities to improve MNG in Kenya exist	17/19 (virtually all)

## Data Availability

The original contributions presented in this study are included in the article. Further inquiries can be directed to the corresponding authors.

## Appendix A

### Appendix A.1. Key Informant Interview (KII) Guide

Purpose of the KII: To examine the status, enablers, and barriers of Multisectoral Nutrition Governance (MNG) in Kenya. MNG is defined as the iterative process of decision-making, policy development, implementation of strategies, monitoring, and accountability aimed at improving nutrition outcomes across various sectors. It includes political commitment and leadership, multisectoral coordination, policy coherence, adequate capacity, sustainable financing, results measurement, monitoring, and accountability all working synergistically.	
**Target group**	
(i) Government staff working in line ministries as program managers or program officers. (ii) Non-government/partner program managers and officers supporting the government to advance the nutrition agenda in Kenya. This includes development partners, United Nations (UN) agencies, Civil Society Organizations (CSOs), private sector organizations, and other non-governmental organizations.	
**Instructions**	
The interviewer will carry out the following: - Start by introducing themself and thank the interviewee for taking time for the interview. - Review informed consent requirements and obtain the interviewer’s consent to participate in the interview. - Request permission to audio record the interview. - Explain the purpose of the interview, the definition of MNG and areas to be covered. - Inform the interviewee that the questions will focus on the ministry they work for or support. - Give a chance to the interviewee to introduce themselves. - Inform the interviewee how long the interview is likely to take (approximately 45–60 min). - Inform the interviewee that the interview is based on their perceptions and they are free to indicate areas they have no information.	
Interviewer details Date: Name: Position:	
**General questions**	
Thank you again for taking the time to meet with me today. I will start by asking general questions before moving to specific questions. 1. Could you please provide a brief overview of your role? 2. Can you tell me a little bit about the structure and functioning of MNG in Kenya *(how does it work)*? 3. What strategies or approaches have been successful in promoting MNG? 4. What are the barriers and challenges hindering effective MNG? 5. In your experience, is there any relationship between multisectoral nutrition governance and outcomes (in the past 10 years)?	
**Main Question**	**Probes**
**A. Political commitment, authority, and leadership**	
1. How would you describe the level of political commitment towards addressing nutrition issues and improving multisectoral nutrition approaches in this ministry?	- What are examples of recent initiatives (policy, institutional, and operational) that demonstrate political commitment towards multisectoral nutrition programming?
2. How would you generally rate the support from ministry leadership in terms of enabling you to oversee and implement nutrition actions?	- Are there any areas where you feel additional support or resources are needed to effectively carry out your responsibilities related to nutrition?
**B. Policy coherence and coordination**	
1. What steps is the ministry taking to ensure nutrition is adequately integrated into its strategies and plans?	- Ask about challenges
2. Describe the extent to which your ministry coordinates and collaborates with other ministries on nutrition-related issues	- What are the challenges or barriers to coordination and collaboration you have encountered?
**C. Transparency and accountability**	
1. Is there clarity regarding who is accountable for achieving nutrition-related targets at all levels? Are roles and expectations communicated regularly?	
**D. Financing**	
1. Could you walk me through the process of how your ministry integrates nutrition considerations into the annual planning and budgeting process?	- How are priorities for nutrition activities determined within the budgeting process? (Is this probe important?)
2. How would you explain the level of budgetary support allocated to nutrition initiatives within your ministry? (Is it sufficient? Are there areas for improvement?)	- How do these allocations compare to other priorities within the ministry? - Are there any challenges related to funding allocation or utilization?
**E. Capacity**	
1. Does the ministry have the necessary technical capacity to support staff to plan and implement nutrition activities effectively?	
2. How does the ministry ensure that staff involved in nutrition planning and implementation are adequately trained and skilled in the relevant areas?	- Are there any areas where additional capacity-building efforts in nutrition are needed?
**F. Results measurement and monitoring**	
1. Can you describe the existing monitoring and evaluation systems and processes for nutrition within your ministry?	- Are the data shared frequently with other ministries? If yes, which information is shared and how often?
2. Are there any challenges or issues related to the systems and quality of nutrition-related data in your ministry?	- How do these challenges affect data quality and utilization?
3. How does your ministry utilize nutrition data to inform your programs and policy decisions?	
**Conclusions**	
Reflecting on our discussion today, is there anything else you feel is important for me to know about the state of MNG in your ministry and in general? Thank you for taking the time for this interview. If you have additional questions or concerns, please feel free to reach out to me or the Principal Investigator.	
